# Processing Rhythmic Pattern during Chinese Sentence Reading: An Eye Movement Study

**DOI:** 10.3389/fpsyg.2015.01881

**Published:** 2015-12-09

**Authors:** Yingyi Luo, Yunyan Duan, Xiaolin Zhou

**Affiliations:** ^1^Department of Psychology, Center for Brain and Cognitive Sciences, Peking UniversityBeijing, China; ^2^Faculty of Science and Engineering, Waseda UniversityTokyo, Japan; ^3^Department of Linguistics, Northwestern UniversityEvanston, IL, USA; ^4^Beijing Key Laboratory of Behavior and Mental Health, Peking UniversityBeijing, China; ^5^Key Laboratory of Computational Linguistics, Ministry of Education, Peking UniversityBeijing, China; ^6^PKU-IDG/McGovern Institute for Brain Research, Peking UniversityBeijing, China; ^7^Collaborative Innovation Center for Language Competence, Jiangsu Normal UniversityXuzhou, China

**Keywords:** prosody, rhythmic pattern, word order, compounding, sentence reading, eye movements, scanpath analysis

## Abstract

Prosodic constraints play a fundamental role during both spoken sentence comprehension and silent reading. In Chinese, the rhythmic pattern of the verb-object (V-O) combination has been found to rapidly affect the semantic access/integration process during sentence reading (Luo and Zhou, 2010). Rhythmic pattern refers to the combination of words with different syllabic lengths, with certain combinations disallowed (e.g., [2 + 1]; numbers standing for the number of syllables of the verb and the noun respectively) and certain combinations preferred (e.g., [1 + 1] or [2 + 2]). This constraint extends to the situation in which the combination is used to modify other words. A V-O phrase could modify a noun by simply preceding it, forming a V-O-N compound; when the verb is disyllabic, however, the word order has to be O-V-N and the object is preferred to be disyllabic. In this study, we investigated how the reader processes the rhythmic pattern and word order information by recording the reader's eye-movements. We created four types of sentences by crossing rhythmic pattern and word order in compounding. The compound, embedding a disyllabic verb, could be in the correct O-V-N or the incorrect V-O-N order; the object could be disyllabic or monosyllabic. We found that the reader spent more time and made more regressions on and after the compounds when either type of anomaly was detected during the first pass reading. However, during re-reading (after all the words in the sentence have been viewed), less regressive eye movements were found for the anomalous rhythmic pattern, relative to the correct pattern; moreover, only the abnormal rhythmic pattern, not the violated word order, influenced the regressive eye movements. These results suggest that while the processing of rhythmic pattern and word order information occurs rapidly during the initial reading of the sentence, the process of recovering from the rhythmic pattern anomaly may ease the reanalysis processing at the later stage of sentence integration. Thus, rhythmic pattern in Chinese can dynamically affect both local phrase analysis and global sentence integration during silent reading.

## Introduction

To group words into meaningful strings with a hierarchical structure, the human language system employs sophisticated rules and constraints at different levels of representation, such as syntactic and semantic agreements (Frazier and Rayner, 1982; MacDonald et al., 1994; McRae et al., 1998). Violations of these constraints lead to impairments in comprehension, resulting in the slowing of reading speed (Van Gompel et al., 2001; Swets et al., 2008; Wu et al., 2014), defective memory of the sentence (Cohen et al., 2001), and ambiguous, unintended or even unrecognized interpretations (Ferreira et al., 2002; Sanford and Sturt, 2002; Ferreira, 2003). Prosody, i.e., the supra-segmental information conveyed in language, is a type of constraint in the language system. Usually recognized in terms of acoustic variations such as pitch, intensity, and duration, prosodic properties constitute a hierarchical structure, i.e., the prosodic structure, which is believed to be independent of, but related to, the surface syntactic structure of a sentence (Nespor and Vogel, 1986; Selkirk, 2011; for more details of the prosodic structure in Chinese, please see Supplementary Material). Although prosodic constraints are considered to play a fundamental role in speech production and comprehension (Scherer et al., 1984; Fodor, 2002), their functions in written language processing have generally been overlooked in the past.

Recent studies have shown that prosodic and especially metrical features at the word level are represented and utilized during sentence reading (Ashby and Rayner, 2004; Lukatela et al., 2004; Ashby and Clifton, 2005; Ashby, 2006; Ashby and Martin, 2008; Huestegge, 2010; Breen and Clifton, 2011, 2013; Luo et al., 2013; Yan et al., 2014). For instance, information regarding the number of syllables in an English word (Ashby, 2006) or the syllabic length of a Chinese word (Yan et al., 2014; Luo et al., 2015) is activated during silent reading, suggesting the use of suprasegmental features irrespective of whether the script is alphabetic or logographic. Another prosodic feature, word stress, has been extensively investigated in the literature since it is essential for defining the rhythm and the metrical structure in languages like English and German. English words with more stressed syllables or with alternative stress patterns lead to longer viewing durations and more costly re-reading during written sentence processing (Ashby and Clifton, 2005; Breen and Clifton, 2011, 2013). When the alternating stress patterns of a word are associated with its ambiguous syntactic categories, reanalysis of the stress pattern would induce processing costs in addition to the syntactic reanalysis (Breen and Clifton, 2013). Similarly, contrastive accents, which phonologically mark the prominent constituents in syntactic and information structure, are also represented during the comprehension of written texts (Stolterfoht et al., 2007). Its placement, however, is influenced by word stress during incremental reading to avoid stress clashes, i.e., sequences of adjacent syllables bearing stress (Kentner, 2012, 2015). The alteration of accents accordingly affects the parsing of local ambiguous structure, as indicated by the re-reading durations of the subsequent disambiguating regions (Kentner, 2012).

The length of the sentence constituents may also subtly but reliably influence the placement of prosodic boundaries. In certain studies (Fodor, 2002; Hirose, 2003; Jun, 2003; Hwang and Schafer, 2009), readers chose one from the two possible interpretations after reading each sentence with syntactic attachment ambiguity. These sentences contained a critical constituent, which was manipulated to be either long or short in terms of the number of syllables or words. Results showed that the constituent length could alter the attachment preference, presumably because a long constituent could spontaneously trigger a subvocal/implicit prosodic boundary, which could imply a syntactic boundary at the same position (but see Jun and Kim, 2004 for a null effect of this manipulation). Using a similar design, Hwang and Steinhauer (2011) recorded Korean speakers' ERP responses and obtained neural evidence for the impact of constituent length upon subvocal prosodic boundary placing. These findings support the Implicit Prosody Hypothesis (Fodor, 1998, 2002), which claims that syntactic parsing can be influenced by implicit prosodic boundary no matter whether it is triggered by a syntactic boundary or by a long phrase.

In Chinese, constituent length influences production and comprehension in a more strict fashion, even determining the grammaticality of certain syntactic structures in both spoken and written language. For example, a verb-object (V-O) combination (V-O phrase) in principle does not allow for an object syllabically shorter than its governing verb. As a consequence, it would feel odd to a Chinese native speaker to hear or read a disyllabic (i.e., two-character) verb combined with a monosyllabic (i.e., one-character) object noun to form a [2 + 1] V-O pattern; yet they have no problems with a [1 + 1], [2 + 2], or [1 + 2] V-O combination expressing the same meaning (see Luo and Zhou, 2010 for more details). For instance, “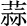, *suan*,” a monosyllabic noun, and “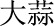, *dasuan*,” a disyllabic noun, have identical meaning “garlic,” and both can form a V-O phrase by monosyllabic verb “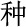, *zhong*, to plant” to express the meaning “to plant garlic.” However, when the verb is replaced by a disyllabic synonym “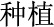, *zhongzhi*, to plant,” only the disyllabic noun “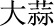, *dasuan*” is allowed to combine with this verb. Since many of these disyllabic and monosyllabic words are just alternative forms of each other, an indication of the flexibility of word length in Chinese (Duanmu, 2000), the oddity of [2 + 1] V-O could not be attributed to the violation of syntactic- or world-knowledge-based constraints. Rather, it has been intensively discussed with respect to “rhythmic pattern” in the Chinese prosodic structure (Feng, 1997; Duanmu, 2000; Wang, 2008; Zhou, 2011). The rhythmic pattern, referring to the combination of words with different numbers of syllables, is considered to constrain lexical selection for different syntactic structures; it is even able to “drive” syntactic operation, causing the alternation of word order (Feng, 1997).

One specific prosodic constraint, namely, the rhythmic pattern of the V-O noun (V-O) combination, has been found to rapidly affect the semantic access/integration process during silent sentence reading (Luo and Zhou, 2010). By manipulating the word length of the verb and by using ERP measures, Luo and Zhou (2010) examined in two experiments the reader's brain responses to a Chinese V-O phrase positioned at the end of a sentence, which could be of either the correct [1 + 1] or the incorrect [2 + 1] pattern. While the V-O was visually presented as a whole phrase with two or three characters on the screen in Experiment 1, the verb and the object were seen consecutively in Experiment 2. Results reliably showed that the [2 + 1] pattern elicited both a larger frontocentral negativity and a larger posterior positivity as compared with the [1 + 1] pattern in the N400 time window after the onset of the whole phrase (Experiment 1) or after the onset of the one-character object (Experiment 2), thus ruling out a visual complexity account for this effect. Moreover, these effects were unlikely to be the carryover of distinct lexical processing of the mono- and disyllabic verbs because there was no remarkable difference in ERP responses to these verbs. Instead, these effects indicated a rapid utilization of the rhythmic pattern during the processing of written sentences: when words are combined to form a phrase, prosody (the rhythmic pattern in this case) proactively imposes constraints on the expectation and selection/access of words that can enter the combination. The violation of the prosodic constraints could initiate reanalysis in a later time window to engender a coherent representation, as reflected by the increased late positivity for the [2 + 1] pattern, relative to the [1 + 1] pattern, 700 ms after the onset of the whole phrase. The findings of N400-like and late positivity effects for the abnormal rhythmic pattern, i.e., the phrasal structure with an inappropriate combination of syllable numbers, were also observed in studies of speech in other languages (Knaus et al., 2007; Magne et al., 2007; Domahs et al., 2008, 2015; Schmidt-Kassow et al., 2015). In these studies, the placement of word stress or syllabic length was manipulated, resulting in correct or abnormal metrical rhythm at the word level. Thus, cross-linguistic evidence seems to indicate common cognitive processing of rhythmic information in languages with divergent default prosodic structures.

Extending our previous work, the aim of the current study was to further investigate how the rhythmic pattern constrains the build-up of phrases or compounds during Chinese sentence reading. Instead of using the ERP measures, here we recorded oculomotor activities while participants read fully visible sentences that contained the critical constituents. The rhythmic pattern was manipulated on the critical V-O with either [2 + 2] or [2 + 1] pattern, and the combination was positioned at the middle of a sentence to avoid the potential influence of sentence-final integration process on local processing. Moreover, such a V-O combination was used to modify a disyllabic noun (e.g., 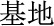, *jidi*, base) to form a new noun compound. As a modifier within a compound, if the verb is disyllabic, the word and the object must be reversed to become a structure as O-V-N (e.g., 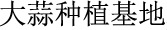, *dasuan zhongzhi jidi*, farm for planting garlic). This phenomenon demonstrates the influence of word length upon morphosyntactic operation in compounding/phrasing (Duanmu, 1997; Feng, 2004). Thus, by crossing rhythmic pattern and word order, we created four types of sentences with the critical noun compounds in the middle of the sentence (see Table 1). The V-O preceding the head noun was either in the V-O order (i.e., an incorrect order for constructing this compound) or in the reversed O-V order (i.e., the correct order). This design allows us not only to examine the similarities and differences between the processing of the rhythmic pattern and word order constraints but also to explore to what extent the prosodic process interacts with the morphosyntactic process in the build-up of sentence representation during reading (c.f., Zec and Inkelas, 1990; Feng, 2004; Eckstein and Friederici, 2005, 2006; Selkirk, 2011).

**Table 1:** **Conditions, interest regions, and exemplar sentences with approximate literal translations**.

**Condition**	**Example**						
		**Region 1**	**Region 2**	**Region 3**	**Region 4**		**Region 5**
RHY+ORD+	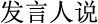	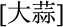	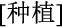	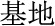	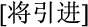	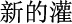	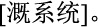
	fayanren shuo	[dasuan]	[zhongzhi]	jidi	jiangyinjin	xinde	guangaixitong
RHY-ORD+	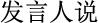	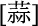	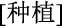	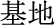	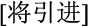	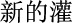	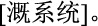
	fayanren shuo	[suan]	[zhongzhi]	jidi	jiangyinjin	xinde	guangaixitong
	spokesman said	[garlic]	[to plant]	district	will introduce	new	irrigation system
RHY+ORD-	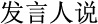	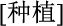	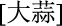	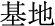	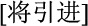	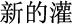	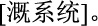
	fayanren shuo	[zhongzhi]	[dasuan]	jidi	jiangyinjin	xinde	guangaixitong
RHY-ORD-	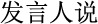	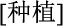	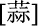	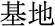	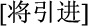	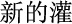	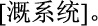
	fayanrenshuo	[zhongzhi]	[suan]	jidi	jiangyinjin	xinde	guangaixitong
	spokesman said	[to plant]	[garlic]	district	will introduce	new	irrigation system
Translation:	The spokesman said the district for planting garlic will introduce new irrigation system						

We also sought to examine the reanalysis process induced when the two types of constraints are violated, with a focus on differential time courses that could be revealed by oculomotor activities (see Rayner, 1998, for a review). In eye-tracking research, the attempt to confirm or revise the initial analysis is defined as reanalysis (Meseguer et al., 2002). Reanalysis could occur at different stages of processing, depending on the type of the linguistic information involved (Boland and Blodgett, 2001; Sturt, 2007). For the current study, we first expected to observe slowdown of reading and more regressive eye movements immediately after the detection of the rhythmic pattern or word order violation; this detection could take place with different time courses. Readers were supposed to be aware of the prosodic anomaly when they finished reading the verb and its object, i.e., the first two words of the compound (Luo and Zhou, 2010), before viewing the head noun. In comparison, the anomaly of word order was unlikely to be detected before the head noun was encountered because the alteration of word order occurred only under the specific circumstance of constructing a compound. Therefore, reanalysis was expected to take place at the second word of the three-word compound for the violation of rhythmic pattern but at the third word (head noun) for the violation of word order. Moreover, we expected that the subsequent reanalyses triggered by these two types of violations would be reflected in temporally dissociated oculomotor measures, given that the effects for prosodic violation (i.e., intonation mismatch) and syntactic violation (i.e., word category or word order violation) were obtained in different time windows in the previous ERP studies (Eckstein and Friederici, 2005, 2006). An alternative prediction would be that the reanalysis of either type of anomaly would mainly affect the late stage of processing according to the findings in Breen and Clifton (2013). Accordingly, we would specifically expect differences in regressive eye movements in response to our manipulations.

In particular, we explored the reanalysis process at a relatively late stage, which has generally been overlooked in the past, i.e., after the reader has finished viewing the sentence at least once. The reader may still entertain the demand to re-read the sentence or to go over its constituents even when he/she finishes reading the last word of the sentence (Meseguer et al., 2002; Sturt, 2007). That is, reanalysis can last through the sentence re-reading stage. Malsburg and Vasishth (2011) pointed out that over half of the sentences engender regressions from the end of the sentence no matter whether the reader was garden-pathed or not. These regressive eye movements tend to illustrate a long-lasting integration process in which all the lexical information has been accessed. Here we not only used the conventional analysis (see Clifton et al., 2007, for a review) to examine the total reading durations for re-reading but also adopted a newly developed method, scanpath analysis, to analyze the pattern of re-reading (Malsburg and Vasishth, 2011). A scanpath refers to a sequential pattern of eye-fixations. Distinct from conventional eye-tracking measures, scanpath analysis integrates both the spatial and the temporal structure of the eye-movements during reading, providing a global view of eye movements' dynamic changes within a space. It would particularly make contributions in studies that have multiple regions of interest and a large amount of long saccades (Mitchell et al., 2008; Malsburg et al., 2014), and be beneficial to the exploration of re-reading. For example, inbound or outbound regressions and second-pass reading times for the pre-defined region(s) are usually measured to reflect the regressive eye movements occurring at the late stage of sentence processing. But these measures, by definition, have the deficits in quantitatively depicting the sequence of serial saccadic movements and the start-and-end of each of them. This information might be peripheral for investigating the initial reading of the sentence because readers are inclined to read region-by-region, from the beginning toward the end. By “initial reading,” we mean the reading processes that occur before the eyes reach the end of the sentence. It nevertheless is critical for re-reading since saccadic span and moving directions are more flexible and optional at such a late stage, and as a consequence, should be more vulnerable to experimental manipulations. In other words, investigating eye movements for sentence re-reading requires approaches with better integration of temporal and spatial information.

During re-reading, we expected to see more integration difficulty for sentences with rhythmic pattern or word order anomaly, relative to normal sentences; this difficulty would be reflected in reading durations and regression on the last words. In addition, the scanpath analysis of eye movements in re-reading would show that the violations have long-lasting influences upon eye movements during re-reading, given that the recovery from anomaly could not be accomplished during the initial reading. Alternatively, if the anomaly could have been fixed during the initial reading, we would expect to observe no difference between normal sentences and anomalies in the scanpath analysis. The latter alternative is possible because, for native speakers, violations of the rhythmic pattern or word order constraints are salient but not “unforgivable”: the reader can easily detect the error right before or on the head noun and compute the correct meaning of the compound based on lexical clues.

## Methods

### Participants

Thirty undergraduate and graduate students (aged between 20 and 28 years, 14 females) from Peking University participated in this experiment. All of them were right-handed, mentally healthy native speakers of Chinese. They had normal or corrected-to-normal vision. This study was approved by the Ethics Committee of the Department of Psychology, Peking University.

### Materials and design

Four critical types of sentences were created (Table 1), each containing 95 sentences with a compound in which a disyllabic verb together with an object noun acted as a modifier of a head noun: correct O-V order with the normal rhythmic pattern (i.e., RHY+ORD+), correct O-V order with the abnormal rhythmic pattern (i.e., RHY−ORD+), incorrect V-O order with the normal rhythmic pattern (i.e., RHY+ORD−) and incorrect V-O order with the abnormal rhythmic pattern (i.e., RHY−ORD−). Each critical sentence was created with the structure “S1 + V1 + S2 +VP2,” with the main subject noun (S1) having 2–3 characters (syllables), the main verb (V1) having 1 character, and its object clause which contained a compound as the subject (S2) and a predicate structure (VP2) with 6–11 characters. Table 1 gives the exemplar sentences for the four conditions.

Ninety-five sets of V-O pairs were selected, with the verb being monosyllabic (e.g., 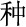 *zhong*, to plant) or disyllabic (e.g., 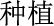, *zhongzhi*, to plant) *zhongzhi*, to plant) and the semantically congruent object noun being monosyllabic (e.g., 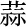, *suan*, garlic) or disyllabic (e.g., 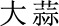, *dasuan*, garlic). Different forms of each pair were synonyms, expressing the same meanings and having the same V-O syntactic relationship; moreover, the character (morpheme) of the monosyllabic word was a constituent of its disyllabic counterpart. The word frequencies of the monosyllabic nouns are generally higher than those of the disyllabic forms, 60 vs. 29 per million according to the Lancaster Corpus of Mandarin Chinese (McEnery and Xiao, 2004) or 42 vs. 17 per million according to SUBTLEX-CH (Cai and Brysbaert, 2010). We selected three kinds of combinations in each set as the experimental stimuli: a monosyllabic verb with a monosyllabic noun, a disyllabic verb with a monosyllabic noun, and a disyllabic verb with a disyllabic noun. For the latter two, word order of the combination was manipulated as either V-O or O-V. Thus, there were five types of pairs, each of which was then combined with a disyllabic noun (e.g., 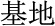, *jidi*, farm, base) to form a compound (see Table 1). This disyllabic noun could only act as the head noun of the compound and could not be viewed as an object of the verb because of the selectional restriction of the verb.

Note that, apart from the four critical conditions, the experiment also included a fifth, unanalyzed condition in which sentences had the same structure and words as the other critical sentences except that the compound took a monosyllabic verb, which was not used in the 4 critical conditions, with a monosyllabic noun as the modifier in a V-O sequence. These correct sentences, 19 in each test list, were taken as fillers in the five lists constructed using a Latin-square procedure. Thus, each test list had 95 critical sentences, 19 for each experimental condition. Another 80 sentences were also added into each list as fillers. They were structurally similar to the critical sentences except that the critical compounds were replaced by constructions like “V-O + *de* + noun,” “O-V + *de* + noun,” and “object + *d*e+ verb + noun” with various types of acceptable rhythmic patterns and/or by compounds with a monosyllabic head. All the fillers were well-formed. Stimuli in each list were pseudo-randomized such that no more than 3 sentences from the same condition would appear consecutively.

### Apparatus

Eye movements were recorded with an EyeLink 2K system at a sampling rate of 2000 Hz. Each sentence was presented in one line at the middle vertical position of a 21-inch CRT screen (1024^*^768 resolution; frame rate 100 Hz). The font *Fangsong*-29 was used, with one character subtending 1 degree of visual angle. Participants read each sentence with their head positioned on a chin rest 78 cm from the screen. All recordings and calibrations were based on the left eye but viewing was binocular.

### Procedures

Participants were calibrated with a nine-point grid. A fixation cross was presented at the position on the screen where the first character of the sentences would appear. The fixation was presented for 500 ms, followed by visual presentation of the whole sentence. Participants were required to silently read the sentence and to press a button on a joystick when finished reading. The button pressing caused the sentence to disappear and a verification sentence to present in about one-third of the trials in the list, including fillers. Participants were instructed to answer, by pressing the “yes” button with their left index finger and the “no” button with their right one, whether the verification sentence was semantically congruent with the preceding sentence. Half of the trials required a “yes” answer and half required a “no” answer. The content of the verification sentence could be related to the overall meaning or to any part of the target sentence; this was to minimize any potential influence on eye movements (particularly the re-reading pattern) by task demand. Specifically, 30 critical trials were followed by the verification sentences. In 18 trials, the head noun of the critical compound, but not the V-O combination, was mentioned in the probe sentence (e.g., “The farm's irrigation system will be changed” for the exemplar in Table 1). In another 12 trials, the probe sentence concerned the comprehension of the V-O combination (e.g., “The planting of garlic needs watering.”). Each trial ended with a rating task on the well-formedness of the sentence. A 2-by-2 grid filled with the numbers 1, 2, 3, 4 respectively would appear on the screen, and participants were instructed to press one of the four corresponding buttons on the joystick to assess the well-formedness of each sentence, with “4” representing that the sentence was not well-formed or the expression was unnatural and “1” representing that the sentence was well-formed and the sentence was expressed in a conventional way. While the verification task required memory and comprehension of the sentence overall as well as parts of the sentence, the well-formedness rating could boost the sensitivity to the unnatural regions. Participants underwent a practice block of 15 trials before the formal experiment.

### Conventional analysis

Five regions were selected as the regions of interest, as shown in Table 1. Region 1 contained the first component of the V-O, composed of 1–2 characters (e.g., the word “to plant” in the example); Region 2 contained the second component of the V-O, which was composed of 1–2 characters (e.g., “garlic”); Region 3 contained the head noun of the critical compound, composed of 2 characters (e.g., “district”); Region 4 contained the component (a verb phrase or an adverbial of the predicate structure in the clause) after the compound, composed of 2–3 characters (e.g., “will introduce”); Region 5 contained the last 3 characters of the sentence (e.g., the last three character of “irrigation system”), except for a few sentences in which only the last two characters were included. The latter was because there were only two characters left after Region 4. Alternatively, defining the last 2 characters as Region 5 for all sentences yielded the same pattern of effects as the one reported in this article; but given that the last two characters were easily parafoveally processed and skipped without fixations, we choose to report the analysis using the 3-character definition for Region 5.

Regression Path Duration (RPD) and the probability of Regression Out (REG) for each region were the measures of instant reanalysis during first reading. RPD was the summed fixation duration from when the region was first fixated until the eyes first moved past the region. REG included the percentage of trials in which at least one regression was made from a given region to previous parts of the sentence prior to leaving that region in a forward direction. First-fixation durations shorter than 60 ms or longer than 800 ms, or Gaze Duration (GD, i.e., the sum of fixation duration from the eyes first entered the region until the eyes moved out) shorter than 60 ms or longer than 1000 ms were excluded from duration and regression analyses, leaving 96% of observations across the five defined regions for statistical analyses.

Estimates were from a linear mixed model (LMM) for durations and a generalized linear mixed model (GLMM) for percent regressions (Baayen et al., 2008), with crossed random effects for participants and items using the *lmer* program of the *lme4* package (Bates et al., 2008) in the R environment for statistical computing (R-Development Core Team, 2009). Due to the large number of trials, the *t*-distribution approximated the normal distribution, and estimates larger than 2 *SE*, i.e., absolute *t*-values (for LMM) or *z*-values (for GLMM) > 1.96 were interpreted as being significant.

### Scanpath analysis

We generally followed the method introduced in Malsburg and Vasishth (2011) to perform scanpath analysis. Scanpath analysis (Cristino et al., 2010; Malsburg and Vasishth, 2011) first quantifies the dissimilarities between every two scanpaths. Similar scanpaths are then clustered and, as a consequence, a prototype of each cluster can be extracted. In this study, each prototype depicted one specific regressive pattern of re-reading. Thus, we were able to both explore what kinds of regressive patterns were mainly triggered for a particular condition and to perform comparisons between conditions by examining the distribution (i.e., percentages) of their scanpaths in each cluster.

The dissimilarities between scanpaths can be measured with “scasim” (Malsburg and Vasishth, 2011), which is a type of global distance between any two fixation sequences. The core idea of this distance is like the edit distance (Levenshtein, 1966), quantifying the dissimilarity of two sequences as the overall penalty to transform one sequence into the other. The pre-defined penalty is a function of locations and durations of fixations in the two sequences:

$$\begin{array}{l} d(f,g)=|dur(f)-dur(g)|\times m^{distance(f,g)}+|dur(f)\\ \;+dur(g)|\times (1-m^{distance(f,g)}). \end{array}$$

In this definition, *f* and *g* refer to any of the fixations from two scanpaths, respectively. The function *dur*() defines the duration of the fixation, the function *distance*() defines the distance in the visual field between *f* and *g*, and *m* is a constant which approximates the drop of visual acuity as the distance increases. As there could be at maximum $C_{m+n}^{n}$ ways to align the fixations given that the two sequences have *m* and *n* fixations respectively, at maximum $C_{m+n}^{n}$ overall penalty values could be generated. The minimal value among them was defined as the scasim between these two sequences.

For the further clustering, it is recommended to map all the scanpaths into a multi-dimensional space while keeping the distances between them undistorted because a coordinate space entertains more options to detect clusters than only distance matrix. The space can be built using the non-metric multi-dimensional scaling (i.e., non-metric MDS, Kruskal, 1964), with the scasim between each pair of them being assigned as their distance. The goodness of fit of this space can be quantified using a residual sum of squares called the stress of a map, which ranges between 0 and 1, and smaller stress means better fit (c.f., Kruskal, 1964). We used a multi-dimensional space instead of a one-dimensional space in that the scanpaths and their dissimilarities may be a consequence of several factors, including experimental manipulations and reading speed, which should be depicted as a variety of dimensions. When a 2- or 3-dimensional space finely represented the data, this scanpath space was visible, although the practical implications of the dimensions were beyond our concern.

Consequently, clustering can be adopted to classify similar scanpaths into the same cluster. Once a cluster is determined, a representative prototype of the constituent scanpaths can be also determined to reflect the pattern features of this cluster. Here we defined the prototype as the one in the cluster's center of gravity, which minimized the dissimilarity to all other constituent scanpaths.

The distribution (i.e., percentage) of scanpaths was then examined as a function of conditions across the clusters. The assumption was that trials of each condition would not be equally assigned to the clusters if the experimental manipulations influence the reading pattern; rather, one cluster may have consisted of more trials from certain condition(s) than the others, and thus distribution pattern in clusters could differ between conditions. For instance, if only two clusters were detected, we could examine the parameters in a logistic regression model in which the cluster classification (two-level) is taken as the response variable (either 0 or 1) and the rhythmic pattern and word order as explanatory variables. If more than two clusters were detected, then a generalized logistic regression with a multinomial response variable, called logit model, could be used instead of logistic regression (Agresti, 2002). The model compares each response category with a baseline category, which is often designated as the most common category with the largest cluster size (Agresti, 2002, p. 268). In addition, a traditional chi-square test was applied as a conservative supplementary to verify the existence of difference between the overall distribution of trials on conditions and the distribution of trials on conditions in a specific cluster.

All data analysis was done in GNU-R (R-Development Core Team, 2009). Scasim dissimilarity of pairs of scanpaths was calculated using the *scanpath* package (Malsburg and Vasishth, 2011). Maps of scanpaths were fit on similarity per fixation scores using the function *isoMDS* from the package *MASS* (Venables and Ripley, 2002). Clusters were detected on this map by fitting mixture of Gaussians models using entropy maximization (Fraley and Raftery, 2002). Calculation of the mixture of Gaussian models was performed using the *mclust* package (Fraley and Raftery, 2007). Finally, we used a logit model (Agresti, 2002) to find out whether the occurrence of clusters and experimental factors were statistically independent, using the function *multinom* from package *nnet* (Venables and Ripley, 2002).

## Results

### Accuracy and rating

On average, participants correctly answered 94.9% (*SD* = 6%) of all the probe questions, indicating that they read the sentences carefully. For the experimental sentences, 30 of them were followed by a probe question referring to the interpretation of the noun compound: the accuracy rate was 95.48% (*SD* = 6%) for the 18 questions concerning the head noun and was 94.30% (*SD* = 9%) for the remaining 12 questions concerning the meaning of the V-O combination. The high accuracy suggests that the critical compounds were well comprehended across all the experimental conditions (see Table 2), although the readers showed the tendency of having higher accuracy for sentences with the abnormal rhythmic pattern than for sentences with the normal rhythmic pattern (by 4.5%, *t* = 1.9). As shown on Table 2, the rating scores of all conditions were less than 2, which may imply that the readers were adopting a relatively loose criterion in judging the well-formedness with respect to the violations of rhythmic pattern or word order. But there were distinctions between the four critical conditions, as confirmed by the statistical analysis with the LMM, which included rhythmic pattern and word order as two within-participant factors. Sentences with abnormal rhythmic pattern were generally rated as more odd than those with normal rhythmic pattern (1.48 for sentences with abnormal rhythmic pattern and 1.69 for sentences with normal rhythmic pattern), *t* = −6.51. Although there was no significant main effect of word order, an interaction between the two factors was found, *t* = 2.3. Further comparisons showed that the abnormal rhythmic pattern led to worse comprehensibility not only when the word order was correct (by 0.27, *t* = 6.65) but also when the word order was incorrect (by 0.13, *t* = 3.26). However, the incorrect word order resulted in worse comprehensibility only when the rhythmic pattern was abnormal (by 0.18, *t* = 4.55), not when the rhythmic pattern was normal (*t* < 1.2).

**Table 2:** **Grand means and standard errors of accuracy rate and well-formedness rating by experimental condition**.

		**RHY+ORD+**	**RHY−ORD+**	**RHY+ORD−**	**RHY−ORD−**
ACC	Type I	96.9% (1.8%)	95.8% (2.1%)	93.8% (2.5%)	96.9% (1.8%)
	Type II	90.0% (3.9%)	98.3% (1.7%)	95.0% (2.8%)	96.7% (2.3%)
	Overall	94.4% (1.7%)	97.2% (1.2%)	94.4% (1.7%)	96.9% (1.2%)
WF		1.39 (0.03)	1.66 (0.03)	1.58 (0.03)	1.71 (0.03)

*ACC, percent accuracy; Type I, probe questions concerning the head noun only; Type II, probe questions concerning the meaning of the whole compound; Overall, overall-average of both question types; WF, well-formedness rating, with 1 represents the best well-formedness in a scale from 1 to 4*.

### Conventional analysis

Measures for all the five regions are shown in Table 3.

**Table 3:** **Grand means and standard errors of eye movement measure by region and experimental condition**.

		**RHY+ORD+**	**RHY−ORD+**	**RHY+ORD-**	**RHY−ORD−**
Region 1	GD	365 (8.38)	304 (7.19)	391 (8.69)	410 (9.41)
	RPD	412 (9.20)	333 (7.90)	462 (10.18)	448 (10.35)
	REG	0 (0)	0 (0)	0 (0)	0 (0)
Region 2	GD	394 (8.49)	439 (10.01)	360 (8.44)	333 (7.85)
	RPD	476 (13.31)	709 (23.13)	429 (11.68)	443 (14.86)
	REG	0.10 (0.01)	0.24 (0.02)	0.12 (0.01)	0.21 (0.02)
Region 3	GD	331 (7.21)	353 (8.48)	337 (6.87)	397 (8.97)
	RPD	400 (12.97)	527 (21.01)	412 (13.59)	627 (21.41)
	REG	0.10 (0.01)	0.18 (0.02)	0.11 (0.01)	0.24 (0.02)
Region 4	GD	396 (9.43)	403 (11.07)	400 (10.32)	401 (10.57)
	RPD	537 (20.22)	581 (22.34)	574 (20.99)	642 (24.91)
	REG	0.11 (0.01)	0.14 (0.02)	0.17 (0.02)	0.19 (0.02)
Region 5	GD	548 (17.03)	531 (17.39)	537 (19.09)	517 (18.01)
	RPD	1113 (36.11)	1056 (34.84)	1155 (36.50)	1062 (37.47)
	REG	0.49 (0.02)	0.46 (0.02)	0.51 (0.02)	0.43 (0.02)

*GD, gaze duration (ms); RPD, regression path duration (ms); REG, regression out probability*.

**Region 1**. Words in this region were disyllabic verbs in the RHY+ORD− and RHY−ORD− conditions and were monosyllabic or disyllabic nouns in the RHY+ORD+ and RHY−ORD+ conditions. Since comparing verbs and nouns may introduce the confounding of word category while comparing disyllabic and monosyllabic words may introduce the confounding of word length, we only compared the disyllabic verbs (e.g., “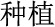 *zhongzhi*,” to plant) for the RHY+ORD− and RHY−ORD− conditions and took rhythmic pattern as a fixed effect factor in the LMM model. No significant difference of duration measures or percentage of regression out probability was found on the verbs between these two conditions.

**Region 2**. In Region 2, we compared the RHY+ORD+ and the RHY−ORD+ conditions, both of which had a disyllabic verb (e.g., “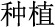 *zhongzhi*,” to plant). We used the same single fixed effect model as we did for Region 1 for the same reason.

RPD on Region 2 was 233 ms longer for sentences with abnormal rhythmic pattern (709 ms) than for sentences with the normal rhythmic pattern (476 ms), *b* = 242.74, *SE* = 23.32, *t* = 10.4. A similar significance pattern was found for GD with an effect of 45 ms. REG was also significantly increased by the abnormal rhythmic pattern (24% for the abnormal condition and 10% for the normal condition), *b* = 1.15, *SE* = 0.19, *z* = 6.13, *p* < 0.001.

**Region 3**. This region included the same disyllabic head nouns (e.g., 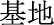, *jidi*, farm, base) for all the four conditions; thus we took rhythmic pattern and word order as two fixed effect factors. Results showed that sentences with the abnormal rhythmic pattern overall yielded longer RPD and a larger REG than sentences with the normal rhythmic pattern, 577 vs. 406 ms for RPD, *b* = 171.2, *SE* = 16.6, *t* = 10.31, and 21% vs. 11% for REG, *b* = 0.91, *SE* = 0.13, *z* = 6.84, *p* < 0.001. Similarly, sentences with incorrect word order had longer RPD as compared with sentences with correct word order, 518 vs. 463 ms, *b* = 61.89, *SE* = 16.6, *t* = 3.73. Such sentences also induced more regressions to previous regions than the sentences with correct word order, 17% vs. 14%, *b* = 0.29, *SE* = 0.13, *z* = 2.2, *p* = 0.028. The same pattern was also obtained in GD analysis.

A significant interaction between rhythmic pattern and word order was found in this region on RPD, *b* = 97.7, *SE* = 33.22, *t* = 2.94, but not on regression probability measures, *p* > 0.1. Further analysis showed that, while RPD showed only a tendency of being longer for sentences with incorrect word order than for sentences with correct word order when rhythmic pattern was normal, *t* < 1.5 or *p* > 0.1, the difference between the two conditions was highly significant when the rhythmic pattern was abnormal, 627 ms for the RHY-ORD- condition and 527 ms for the RHY−ORD+ condition, *b* = 114.13, *SE* = 27.53, *t* = 4.15. This interaction could also be interpreted in terms of the effect of rhythmic pattern as a function of word order. When the word order was correct, sentences with abnormal rhythmic pattern yielded longer RPD (527 ms) than sentences with normal rhythmic pattern (400 ms), *b* = 125.03, *SE* = 23.46, *t* = 5.33; when the word order was incorrect, the difference was even larger (627 ms for the RHY-ORD- condition and 402 ms for the RHY+ORD− condition), *b* = 221.29, *SE* = 22.93, *t* = 9.65.

To summarize, on the head noun, the rhythmic pattern effect emerged regardless of whether the word order was correct or not; however, the size of the effect was larger in sentences with incorrect word order.

**Region 4**. In this region, the abnormal rhythmic pattern led to not only longer RPD (611 ms for sentences with abnormal rhythmic pattern and 555 ms for sentences with normal rhythmic pattern), *b* = 56.21, *SE* = 20.06, *t* = 2.8, but also a higher REG (17% for sentences with the abnormal rhythmic pattern and 14% for sentences with the normal rhythmic pattern), *b* = 0.29, *SE* = 0.13, *z* = 2.22, *p* = 0.026. On the other hand, the violation of word order also resulted in longer RPD (608 ms for sentences with the incorrect word order and 559 ms for sentences with the correct word order), *b* = 46.77, *SE* = 20.06, *t* = 2.33, as well as a higher REG (18% for sentences with incorrect word order and 13% for sentences with the correct order), *b* = 0.51, *SE* = 0.13, *z* = 3.32, *p* < 0.001. Interaction between rhythmic pattern and word order did not reach significance, *t* < 1.0. No significant results were observed on GD, *t* < 0.5.

**Region 5**. Compared with sentences with acceptable rhythmic pattern, sentences with abnormal rhythmic pattern induced *shorter* RPD (1059 ms for sentences with the abnormal rhythmic pattern and 1134 ms for sentence with the normal rhythmic pattern), *b* = −74.69, *SE* = 31.21, *t* = −2.39, and a *reduced* REG (45% for sentences with the abnormal pattern and 50% for sentences with the normal pattern), *b* = −0.24, *SE* = 0.1, *z* = −2.43, *p* = 0.015. Neither the main effect of word order nor the interaction between word order and rhythmic pattern was significant, *ts* < 1. Again, no significant results were observed on GD, *t* < 1.4.

### Scanpath analysis

Participants executed regressions from the last word in 1292 trials of all 2250 trials: 327 for the RHY+ORD+ condition, 335 for the RHY−ORD+ condition, 333 for the RHY+ORD− condition, and 297 for the RHY-ORD- condition. Although 51 trials (3.9%) seemed unusual since their converted distances to all other scanpaths were over three standard deviations larger than those of the other scanpaths, they were kept in the following analysis as removing them did not change the pattern of results.

**Figure 1:**
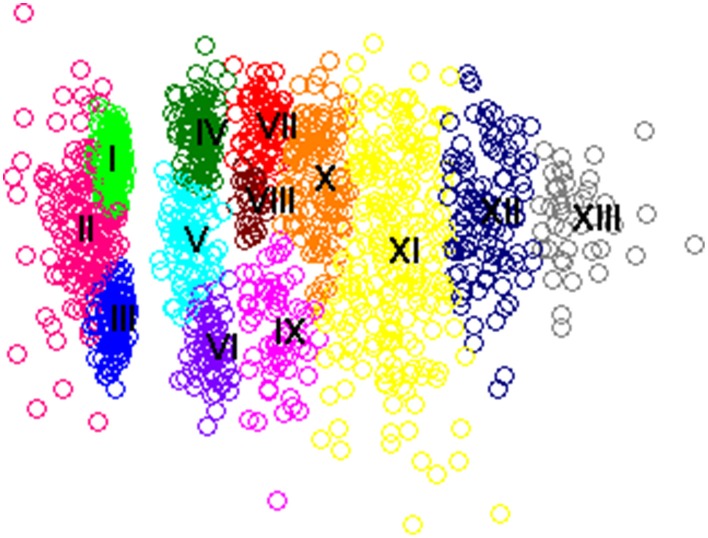
**Map of all regression patterns in the data set originating from region 5**. Colors indicate clusters that were found using mixture of Gaussian modeling. The roman numbers mark the positions on the center of these clusters.

**Figure 2:**
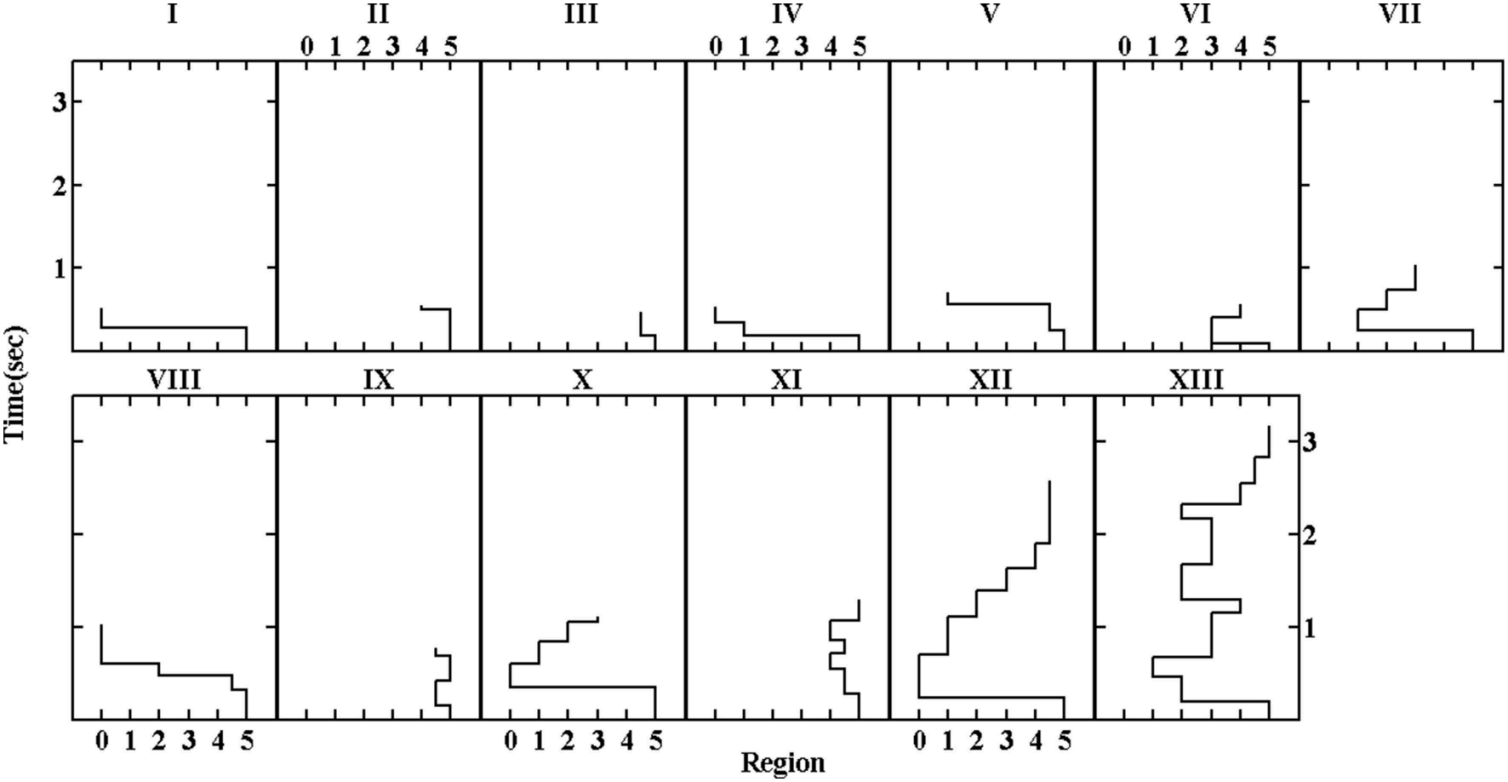
**The regression patterns that were closest to the gravity center of the clusters identified on the 2-dimensional map of all regressions from the data set (see Figure 1), called the prototypical regressive patterns for a cluster**.

We first built a 2-dimension map due to the simplicity and visibility of this model. The stress of this map was 13.32%, indicating that this map was good enough for our purpose (c.f., Kruskal, 1964; Malsburg and Vasishth, 2011). Thirteen clusters were detected on this map using the mixture of Gaussian modeling, which is able to identify the clusters even if they intersect or overlap. Figure 1 shows the map and Figure 2 shows the prototype of each cluster. To better illustrate the locations of the fixations in the scanpaths in terms of sentence structure, we matched the coordinates of location and the critical regions, as shown in Figure 2. Clusters were sorted according to the complexity of constitutional scanpaths.

Scanpath patterns in Figure 2 could be roughly classified into three groups in terms of durations and complexity. Clusters such as Cluster I, II, and III contained simple scanpaths with a single fixation shorter than 400 ms. Clusters IV, V, VI, and VII manifested relatively efficient regressions targeting the compound region, as the scanpaths consisted of 2–4 fixations on the compound region for a total of 600–1000 ms. The other clusters (Clusters VIII–XIII) could be viewed as complex regression patterns, with durations longer than 1000 ms and diversified spatial trajectories. Detailed descriptions of clusters according to the prototypes are as follows.

Simple Cluster I: Regressing from the end of the sentence, with only a fixation located on the beginning of the sentence, including Region 1 and the words before Region 1.

Simple Cluster II: The single fixation on the scanpath mainly dwelled on Region 2, 3, or 4, which was within or next to the critical compound in the middle of the sentence. But the whole scanpath lasted no more than 200 ms, relatively shorter than the single fixations compared with other simple clusters.

Simple Cluster III: The single fixation located on the middle and the later part of the sentence, including Region 4 and words between Region 4 and 5.

Moderate Cluster IV: This cluster consisted of a backward-moving pattern with the first fixation on Region 1 or 2 (i.e., the critical V-O) and the subsequent fixations on positions even closer to the beginning of the sentence. Scanpaths usually lasted around 600–800 ms.

Moderate Cluster V: This cluster also consisted of scanpaths of 600–800 ms with two fixations going backward toward the preceding part of the sentence. A regressive saccade was usually launched from the end of the sentence to Region 4 first, and then to Region 2 or 3 within the critical compound.

Moderate Cluster VI: Scanpaths of around 800 ms with 3–4 fixations. Critical words between Region 2–4 first attracted the regressive saccades from the end of the sentence. A forward saccade then followed, landing at Region 4 or 5.

Moderate Cluster VII: Containing more than three fixations with a total duration of about 900 ms, scanpaths of this cluster showed a dichotomy of saccadic direction. Fixations first reached Region 1, and the subsequent fixations were located either backwards toward the beginning of the sentence (i.e., Region 1 or before), or forwards toward Region 2 and 3.

Complex Cluster VIII: The average duration of the scanpaths was about 1000 ms. The cluster was recognized by its consecutive backward saccades, landing first at the later part of the sentence (i.e., Region 4 or after) and then showing successive stepping toward the beginning of the sentence within 4–5 fixations.

Complex Cluster IX: Scanpaths in this cluster contained four or five fixations and lasted around 1000 ms. Most scanpaths resided in Region 4 and 5.

Complex Cluster X: With 5 or more fixations and with a duration longer than 1000 ms, scanpaths of this cluster also showed a dichotomy of saccadic direction after a long saccade to the early part of the sentence such as Region 1 or 2. The subsequent fixations moved either forwards or backwards.

Complex Cluster XI: In this cluster, scanpaths lasted more than 1500 ms and showed great diversity in terms of saccadic patterns. Some of them mainly dwelled on Region 4 and 5, while others on the middle of the sentence such as Region 2 and 3.

Complex Cluster XII: This cluster consisted of scanpaths lasting longer than 1800 ms with more than 8 fixations. More notably, this cluster clearly manifested the pattern of re-reading the whole sentence from the beginning, with the first fixation located on the beginning of the sentence followed by consecutive forward movements until the end of sentence.

Complex Cluster XIII: Scanpaths with extremely long durations (more than 2800 ms) and diverging saccadic directions were depicted. Most fixations were focused on the middle of the sentence particularly the critical compound (Region 1–3).

Table 4 shows the number of scanpaths in categories, i.e., the distribution pattern of the scanpaths in each cluster by condition. A chi-square test was taken to examine whether the distribution of conditions in each cluster was comparable to the distribution of conditions in all trials. Results showed that Moderate Cluster VI significantly differed from the overall distribution, χ^2^ = 11.57, *p* < 0.003, *df* = 3, while Simple Cluster I and Cluster II were marginally significant, χ^2^ = 7.21, *p* = 0.066, *df* = 3, and χ^2^ = 7.38, *p* = 0.061, *df* = 3, respectively.

**Table 4:** **Count of scanpaths by cluster and condition (2-dimensional map)**.

	**RHY+ORD+**	**RHY−ORD+**	**RHY+ORD−**	**RHY−ORD−**	**Total**
Cluster I	25	39	24	37	125
Cluster II	48	30	50	31	159
Cluster III	24	30	21	27	102
Cluster IV	28	28	23	26	105
Cluster V	18	27	25	18	88
Cluster VI	22	14	27	7	70
Cluster VII	20	24	13	12	69
Cluster VIII	6	7	8	9	30
Cluster IX	19	19	11	15	64
Cluster X	28	31	28	30	117
Cluster XI	45	52	71	52	220
Cluster XII	33	25	21	23	102
Cluster XIII	11	9	11	10	41
Total	327	335	333	297	1292

But chi-square test did not provide the estimates regarding which and how manipulated factors contributed to the significant conditional differences. To address this problem, multinomial logistic regression was further carried out. Cluster XI was chosen in the regression model as the baseline category because it had the largest cluster size among all clusters. Results showed that Simple Cluster I and Moderate Cluster VI were significantly influenced by the violation of rhythmic pattern (Cluster I: *z* = 2.25, *p* = 0.025; Cluster VI: *z* = −2.33, *p* = 0.020). Compared with the normal sentences, sentences with the abnormal rhythmic pattern were associated with more scanpaths of simple patterns and with fewer scanpaths of complex patterns during re-reading. Neither the main effect of word order nor the interaction of rhythmic pattern and word order were significant^1^.

**Figure 3:**
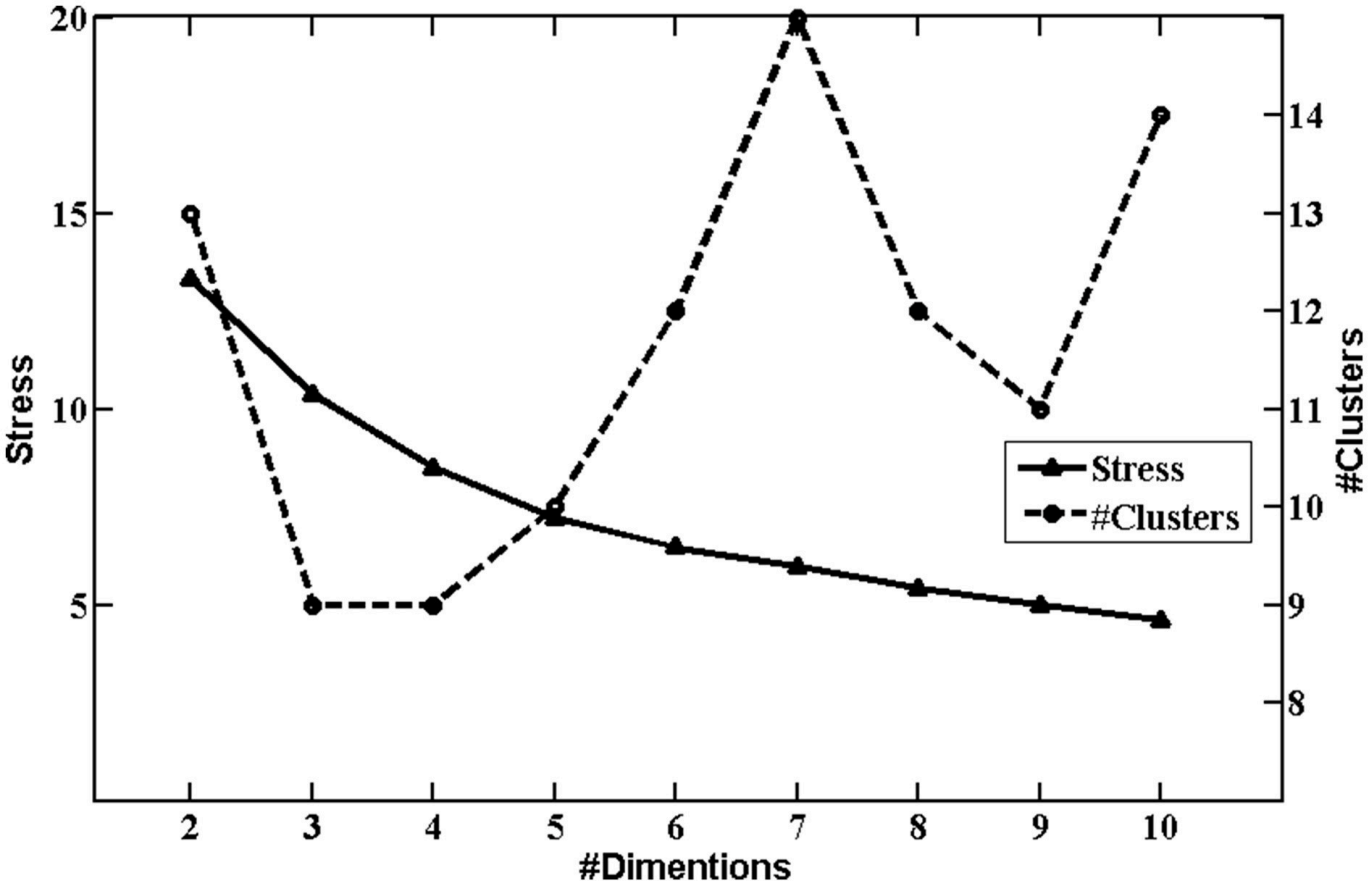
**Stress values and numbers of clusters for increasing numbers of map dimensions**. As the number of dimensions goes up, the stress of maps decreases, i.e., more variance is explained by higher-dimensional maps.

**Figure 4:**
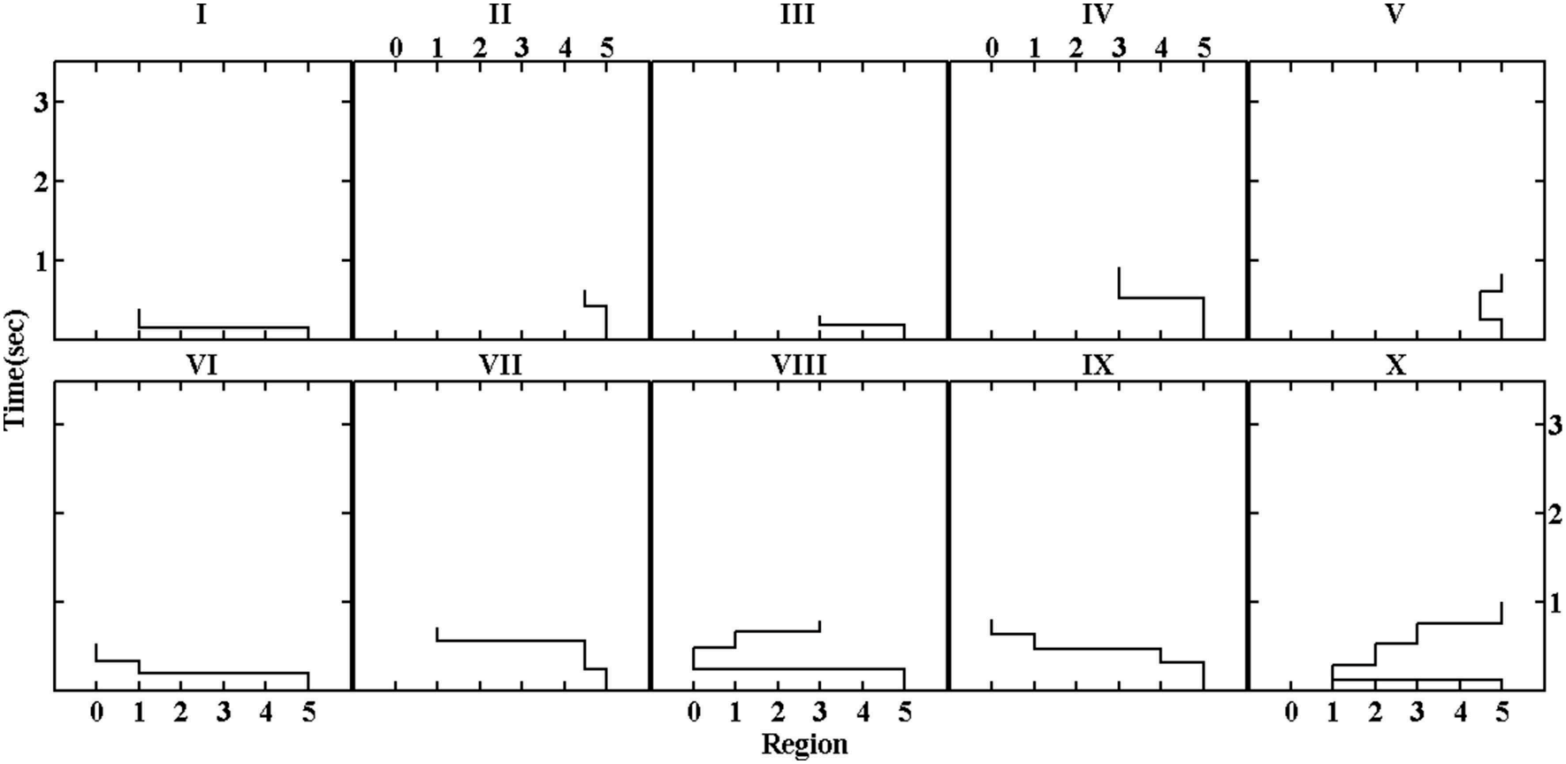
**Prototypical regressive patterns of the clusters on the 5-dimensional map**.

In order to test the reliability of the scanpath classification and to validate the result found on the 2-dimensional space, we also fitted maps for 2–10 dimensions and calculated clusters models for each of them. Figure 3 shows the stress of those maps and the number of clusters obtained as a function of the number of dimensions. The stress (or variance not represented by the map) decreased as the dimension of the map increased, while the number of clusters reached a plateau of around 10 after dimension of the map became large enough. To contrast the 2-dimensional model with a more complex one, we chose the clustering on the 5-dimensional map for further analysis since the 5-dimensional model approximated to the knee in the stress curve which methodologically (or structurally) indicated the possible dimensionality of the data. Figure 4 shows the prototypical scanpaths of the clusters and Table 5 shows the count of scanpaths by cluster and condition.

**Table 5:** **Count of scanpaths by cluster and condition (5-dimensional map)**.

	**RHY+ORD+**	**RHY−ORD+**	**RHY+ORD−**	**RHY−ORD−**	**Total**
Cluster I	26	42	25	37	130
Cluster II	21	13	23	18	75
Cluster III	8	26	11	19	64
Cluster IV	37	18	34	21	110
Cluster V	31	28	39	20	118
Cluster VI	24	27	24	26	101
Cluster VII	19	21	24	16	80
Cluster VIII	42	48	33	41	164
Cluster IX	37	32	39	35	143
Cluster X	82	80	81	64	307
Total	327	335	333	297	1292

Multinomial logistic regression with Cluster X as the baseline category yielded a significant difference between the numbers of trials with the normal and abnormal rhythmic patterns, *z* = 2.03, *p* = 0.04 for Cluster I, and *z* = 1.89, *p* = 0.06 for Cluster III, respectively. As these two clusters reflected simple scanpath patterns, this result indicated that re-reading sentences with the abnormal rhythmic pattern induced more, rather than less, simple regressive eye movements as depicted by cluster I and III. This finding is consistent with the findings of the analysis with the 2-dimensional map which showed increased trials of simple patterns but fewer trials of complex patterns for prosodic violation.

## Discussion

By manipulating the rhythmic pattern and word order of the compound, the present study showed that, during the initial reading of the sentence, both types of violations elicited immediate reanalysis locally within the compound, as reflected by longer reading times and more regressions. These effects also extended to the reading of the region right next to the critical compound. But the interaction, which suggested more demanding reanalysis for double violations than for single ones, only occurred on the head noun of the compound, not on the spillover region. After all words of the sentence had been accessed, however, readers tended to initiate less reanalysis for sentences with the abnormal rhythmic pattern than for sentences with the normal pattern, as shown by shorter re-reading times on the sentence-final words as well as fewer and simpler regressive eye movements. In the following discussion, we start with the issue of processing word order information, and then focus on the immediate use and the late influence of rhythmic pattern information during sentence reading. We note especially the similarities and dissimilarities between the processing of different types of information.

The effect of word order violation was in line with a great number of previous studies reporting prolonged viewing durations and more regressions for sentences with ambiguities or errors (for a review, see Rayner, 1998; for the study on Chinese, see Shi et al., 2000; Zhang et al., 2000, 2002; Hsieh et al., 2009). In particular, the violation of word order was immediately detected at the head noun, resulting in an increase of gaze duration, which is commonly considered to indicate the difficulty of lexical access during sentence reading (Rayner, 2009; Yan et al., 2014). More regressive saccades were then launched toward the preceding regions, reflecting the attempt to integrate the current word into the upstream context (Spivey and Tanenhaus, 1998; Boland and Blodgett, 2001). Such reanalysis continued even to the next region, which can be interpreted as the spillover of integration demands for the previous anomaly (Rayner et al., 1989; Rayner, 1998). However, for the abnormal word order, there seemed to be neither lexical access difficulty nor enhanced reanalysis at the end of the sentence and on re-reading, where sentences with the word order violation were read as fast and smooth as sentences with no word order violation. Results of the offline tasks also showed no impairment of comprehensibility by the single violation of word order, implying that the rapid local reanalysis at the compound was very effective in repairing the word-order-induced morphosyntactic anomaly.

Similar to the erroneous word order, the abnormal rhythmic pattern also elicited the local and immediate reanalysis, but the anomaly was detected earlier on the critical verb of the compound as predicted: a disyllabic verb on Region 2 following a monosyllabic noun received more prolonged gaze durations than the same verb following a disyllabic noun (RHY−ORD+ vs. RHY+ORD+). This finding is inconsistent with some previous eye-tracking studies which observed no effect on first-pass reading times for stress errors and which suggested relatively delayed responses to inappropriate prosodic representations (Kentner, 2012; Breen and Clifton, 2013). However, supporting evidence for a sensitive detection and rapid reanalysis process triggered by prosodic violations comes from a number of ERP studies on rhythmic pattern during Chinese sentence reading (Luo and Zhou, 2010) and on other metrical (Knaus et al., 2007; Magne et al., 2007; Schmidt-Kassow and Kotz, 2009; Rothermich et al., 2010) and phrasal/sentential prosodic properties (Eckstein and Friederici, 2005, 2006) during speech processing. In those studies, prosodic violations led to increased negativities in the N400 time window, i.e., around 300–500 ms after the onset of the critical stimuli, which were considered to reflect a general error detection mechanism (Rothermich et al., 2010) or an early role of prosodic cues in lexical access and meaning integration (Eckstein and Friederici, 2005; Magne et al., 2007). Together with these studies, the current finding of longer gaze duration on Region 2 for the abnormal rhythmic pattern demonstrates the immediate process for detecting the mismatching prosodic features and for resolving the difficulty in lexical access. This process was independent of the word/phrase position in the sentence and the word order of the constituent verb and noun, which was O-V in the present study and V-O in Luo and Zhou (2010). It is possible, however, that such an early effect might be absent if the detection of inappropriate prosodic representation relies on reanalysis triggered by the more complex syntactic garden-path, which requires more elaborative processing (e.g., Kentner, 2012; Breen and Clifton, 2013).

Note that, effects for the abnormal rhythmic pattern cannot be simply explained away as being due to the absence of co-occurrence between the disyllabic verb and the monosyllabic object noun. Abnormal and normal rhythmic patterns used essentially the same words, differing only on one morpheme (see the RHY+ and RHY– conditions in Table 2). More importantly, if the effects were due to the absence of co-occurrence, one would expect to observe similar effects for different types of violations in either oculomotor or ERP responses, an expectation not confirmed by the findings in either the present study or Luo and Zhou (2010). Orthographical or segmental differences between the disyllabic noun in the RHY+ORD+ condition and the monosyllabic noun in the RHY−ORD+ condition could not provide a tenable account for the rhythmic pattern effects either, since the effects were observed on the verb and regions downstream, which were visually and phonologically undifferentiated between conditions. Moreover, this account would predict prolonged viewing times for the combinations with normal rhythmic pattern (2-character noun) than those with abnormal rhythmic pattern (1-character noun) during the first pass reading (Rayner and Raney, 1996; Wang et al., 1999) or for regions (Regions, 2, 3, and 4) following the lower frequency 2-character noun than following the higher frequency 1-character noun, apparently contradicting what we observed in this study.

Local and immediate reanalysis of the abnormal rhythmic pattern also manifested itself as more regressive eye movements launched from Region 2 before reading on, presumably for further confirmation of the perceived information and for repair of the mismatching prosodic structure. Similar patterns were further observed on the subsequent head noun of the compound regardless of whether the word order was correct or not. The prosodic violation caused difficulty in lexical access for the unambiguous head noun of the compound, suggesting that the expectation toward the target word based on rhythmic pattern, which would normally facilitate the processing of the upcoming word, was disrupted. But after carrying out the reanalysis for the whole compound, readers seemed to no longer suffer from failing to generate the incremental lexical expectation, as indicated by the null effect on gaze duration in post-compound Region 4, although increased regressive eye movements remained for reanalysis of contextual integration.

Unexpectedly, when readers continued on and approached the end of the sentence for the first time, the preceding rhythmic pattern violation seemed to facilitate, rather than interfere with the later stage of the sentence comprehension. This observation was confirmed by both approaches of data analysis: the conventional measures revealed shorter re-reading durations on Region 5 and fewer regressions launched from the sentence-end, and the scanpath analysis revealed simpler patterns of eye movements in re-reading sentences. Moreover, the offline comprehension task suggested that the sentences with the abnormal rhythmic pattern were in fact slightly better comprehended, with higher response accuracies, than the correct sentences and the sentences with word order violation.

The later facilitatory effect of the abnormal rhythmic pattern was obviously contradictory to our predictions grounded on previous findings for reanalysis of prosodic or syntactic structure. Reanalysis of word stress during silent reading led to longer second-reading times when it was incurred by reanalysis of syntactic structure (Kentner, 2012; Breen and Clifton, 2013). Incongruent prosody in speech could influence the late components of ERP responses to the critical spoken words positioned near the end of the sentence (Eckstein and Friederici, 2005; Magne et al., 2007). These findings tend to (but not exclusively) favor the claim that the late stage of sentence processing would be impaired by prosodic violations. On the other hand, although Sturt (2007) reported that an ambiguous word with lexical bias in the middle of the sentence would lead to reduced first-pass reading times on the sentence-final regions, it would also lead to more regressions out of the final regions, suggesting a larger rather than a diminished demand of re-reading. A possible account for the current finding is that when encountering a rhythmic pattern anomaly, the reader is likely to process the incoming information with efforts greater than is necessary, i.e., “above and beyond” what should be devoted in normal reading. This engenders not only a recovering process for the anomalous compound but also a more careful and intensive integration for constituents in the sentence, as indicated by longer reading times and more regressions before the sentence-end during the initial reading. This procedure is effective in that the reader is able to build up a clear and coherent representation for the sentence, and less effort is needed for the overall integration during the later stage of reading. In short, when confronted with the abnormal rhythmic pattern that needs to be fixed, the reader may deploy a dynamically adjusted global strategy that starts from sufficient and deep processing (i.e., “Above and Beyond” approach), rather than satisficing and shallow processing (i.e., “Good Enough” approach proposed by Ferreira, 2003).

Another unexpected finding for rhythmic pattern was on its interaction with word order. As predicted, we observed the interaction between the two types of information on RPD of the head noun, indicating that the recovery from double violations was particularly resource-consuming relative to the simple addition of the recovery from either type of violation. This interaction hinted that the rhythmic pattern and word order of the V-O combination were processed and used interactively to contribute to compounding. However, different from the previous research suggesting a mild moderation of prosodic properties on syntactic reanalysis when both prosodic and syntactic errors were detected on the same word (Eckstein and Friederici, 2005, 2006), the present study revealed that repairing the word order violation was minor when the rhythmic pattern was normal but became much more remarkable when the rhythmic pattern was also incorrect (2 ms vs. 100 ms for RPD). In other words, the prosodic structure of the V-O combination strongly affected the processing of word order information during the structuring the compound. Given that the violation of rhythmic pattern was encountered before the head noun, namely, earlier than the detection of word order, it is possible that the prosody-induced, delayed processing of the head noun significantly interfered with the repair of word order which required cues related to lexical and compound meanings.

Given these unexpected findings for rhythmic pattern, how should we compare the current study with previous research? Do they merely suggest the unique processing for a specific prosodic property, or do they point to more general but subtle mechanisms in language comprehension? We believe that our findings may provide a possible perspective for interpreting the similarities and dissimilarities between different types of information, i.e., the recoverability when a certain constraint is violated. Indeed, manipulations of syntactic information usually have significant impacts upon the buildup of the main predicate structure of the sentence and even cause harm to the comprehension (Frazier and Rayner, 1982; MacDonald et al., 1994; McRae et al., 1998; Christianson et al., 2001; Ferreira et al., 2001; Sanford and Sturt, 2002). Similarly, the prosodic properties that were investigated in the previous reading studies could also lead to a syntactic garden-path if mistakenly represented (Kentner, 2012; Breen and Clifton, 2013). By contrast, the rhythmic pattern here does not remarkably affect the representation at the sentential-meaning level in the given context, presumably because the relatively intact visual cues, with only one semantically redundant morpheme missing from the original, correct V-O combination, would suffice for lexical access and syntactic parsing. The “Above and Beyond” approach is assumed to be applied to recover from the rhythmic pattern anomaly which is less damaging to parsing than some other prosodic properties studied so far. On the other hand, the abnormal rhythmic pattern occurred in the earlier part of the sentence in the present study. Compared to the violations detected at the latter part of the spoken sentence in the previous ERP studies (e.g., Eckstein and Friederici, 2005; Magne et al., 2007; Rothermich et al., 2012), the anomaly in this study could bear a longer recovery process by the end of the sentence, as indicated by the increased regression-path durations on Regions 2, 3, and 4. In other words, under certain circumstances we should be able to observe facilitation in the late stage of sentence processing for prosodic violations other than rhythmic pattern, a prediction that could be tested in further studies.

In summary, by manipulating rhythmic pattern as well as word order in compounding and by recording eye movements during silent sentence reading, we observed different reanalysis patterns for the two types of violations. While the reanalysis of both types of information occurs immediately after the detection of errors during the first reading of the sentence, the more effortful recovering process for the abnormal rhythmic pattern at the early stage may ease sentential integration at the later stage of sentence comprehension.

### Conflict of interest statement

The authors declare that the research was conducted in the absence of any commercial or financial relationships that could be construed as a potential conflict of interest.

## Footnotes

^1^We also performed the multinomial logistic regression model with cluster XIII as the baseline category since this cluster had the most similar distribution to the overall distribution of trials according to the result of chi-square test (with the minimum χ 2 value 0.33). Cluster VI showed a robust effect, *z* = 2.06, *p* < 0.05. Test on Cluster I was not significant though, *p* = 0.29.
