# Supplementary material for: Processing Rhythmic Pattern during Chinese Sentence Reading: An Eye Movement Study
Source: Front Psychol. 2015 Dec 9;6:1881. doi: 10.3389/fpsyg.2015.01881 (PMC4673344; doi:10.3389/fpsyg.2015.01881)
Supplement: Supplementary file 1 [file Table1.DOCX]

Supplementary Materials for “Processing rhythmic pattern during Chinese sentence reading: An eye movement study”

Linguistic rhythm is usually defined as the regular alternations between strong and weak, stressed and unstressed syllables (Liberman and Prince, 1977). In many languages, word stress is easy to feel by the native speakers and is reliably captured by phonetic measurements (Fry, 1958). However, linguists disagree on whether Chinese has word stress, and if so, where it is (Chao, 1968; Duanmu, 2000; Selkirk and Shen, 1990; Wang, 2008). No convergent evidence has shown that Chinese native speakers can consistently perceive the relative stress among full-syllable words (Wang, 2008). Neither acoustic analysis of speech found prominent physical cues for word stress (Lin, Yan and Sun, 1984; Wang and Wang, 1993).

Although stress plays an ambiguous role in Chinese metrical structure, there is a consensus that the basic rhythmic unit of Chinese (equivalence of “foot”) is a structure with two-to-three syllables (Chen, 2000; Duanmu, 2000; Feng, 1998; Shih, 1986; Wang, 2008; Zhou, 2011), each of which tends to be isochronally distributed. Chinese native speakers perceive boundaries between two basic rhythmic units but not within them. Rhythm in Chinese, therefore, is considered to indicate that the supra-segmental features, e.g., intensity, duration, pitch, or their combinations, reiterate at equal points in time (Wang, 2008). It is also in line with a more general definition of rhythm given in Gibbon (2015), “Rhythms are temporally regular iterations of events which embody alternating strong and weak values of an observable parameter”.

Both Selkirk (1984) and Nespor and Vogel (1986) proposed a layer of “ phonological word” for the prosodic hierarchy; this phonological word is determined by the lexical word boundary and the foot structure. Chinese prosodic hierarchy lacks such a layer due to the ambiguous word stress as well as the unclear word boundary. A single “字 *zi*” (character) in Chinese usually assumes a syllable in sound form, a unit of strokes with clear visual boundaries in orthographical form, and also a morpheme with independent meaning (Chao, 1968).

**References**

Chao, Y. R. (1968). *Language and symbolic systems*. (CUP Archive).

Chen, M. (2000). *Han yu fang yan de lian xu bian diao mo shi (Tone sandhi: Patterns across Chinese dialects)*. (in Chinese). (London: Cambridge Press).

Duanmu, S. (2000). *The Phonology of Standard Chinese*. (New York: Oxford University Press).

Feng, S. (1998). Lun han yu de “zi ran yin bu” (A discussion of “natural step” in Chinese). (in Chinese). *Zhongguo Yuwen* 1, 40-47.

Fry, D. B. (1958). Experiments in the perception of stress. *Lang. Speech*. 1(2), 126-152.

Gibbon, D. (2015). 2 Speech rhythms–modelling the groove. In *Rhythm in Cognition and Grammar: A Germanic Perspective*, ed. Vogel, R., and Vijver, R. (Walter de Gruyter GmbH and Co KG). 53-80.

Liberman, M., and Prince, A. (1977). On stress and linguistic rhythm. *Linguist. Inq.* 249-336.

Lin, M., Yan, J. Z., & Sun, G. H. (1984). Beijinghua liangzizu zhengchang zhongyin de chubu shiyan (Preliminary Experiments on the Normal Stress in Beijing Disyllables). (in Chinese). *Fangyan*, 1984, 57-73.

Nespor, M., and Vogel, I. (1986). *Prosodic phonology*. (Dordrecht, The Netherlands: Foris).

Selkirk, E. O. (1984). *Phonology and syntax*. (Cambridge, Mass: MIT Press).

Selkirk, E., and Shen, T. (1990). Prosodic domains in Shanghai Chinese. In *The Phonology-Syntax Connection*, ed. Inkelas, S., and Zec, C. 313-337.

Shih, C. (1986). *The phonetics of the Chinese tonal system*. Technical memorandum. AT & T Bell Laboratories.

Wang, H. (2008). *Han yu fei xian xing yin xi xue (The nonlinear phonology in Chinese).* (in Chinese). (Beijing: The Peking University Press).

Wang, J. and Wang L. (1993). Pu tong hua duo yin jie ci yin jie shi chang fen bu mo shi (The distribution pattern of the syllable duration of Mandarin polysyllabic words). *Zhongguo Yuwen* 2, 112-116.

Zhou, R. (2011). *Xian dai han yu yun lv yu yu fa de hu dong guan xi yan jiu (Research on the interactive relationship between rhythm and grammar of modern Chinese)* (in Chinese). (Beijing: The Commercial Press).
